# Nomogram Predicts Overall Survival in Patients With Stage IV Thyroid Cancer (TC): A Population-Based Analysis From the SEER Database

**DOI:** 10.3389/fonc.2022.919740

**Published:** 2022-07-11

**Authors:** Tianqing Yang, Tingting Hu, Mingyi Zhao, Qingnan He

**Affiliations:** Department of Pediatrics, Third Xiangya Hospital, Central South University, Changsha, China

**Keywords:** Thyroid cancer (TC), stage IV, nomogram, overall survival (OS), prognostic model

## Abstract

**Background:**

Stage IV Thyroid cancer (TC) has a relatively poor prognosis and lacks a precise and efficient instrument to forecast prognosis. Our study aimed to construct a nomogram for predicting the prognosis of patients with stage IV TC based on data from the SEER programme.

**Methods:**

We enrolled patients diagnosed with TC from 2004 to 2015 in the study. Furthermore, the median survival time (MST) for the patients equalled 25 months. The patients were split into two groups: the training group and validation group. We used descriptive statistics to calculate demographic and clinical variables, Student’s t test was used to describe continuous variables, and the chi-square test was used to describe classified variables. We used the concordance index (C-index) to evaluate discrimination ability and calibration plots to evaluate calibration ability. The improvement of the nomogram compared with the AJCC TNM system was evaluated by the net weight classification index (NRI), comprehensive discriminant rate improvement (IDI) and decision curve analysis (DCA).

**Results:**

There were 3501 patients contained within our cohort, and the median follow-up was 25 months [quartile range (IQR): 6-60] in the whole population, 25 months (IQR: 6-60) in the training cohort, and 25 months (IQR: 5-59) in the validation cohort. The C-index value of the training cohort equalled 0.86 (*95% CI: 0.85-0.87*), and the value of the validation cohort equalled 0.85 *(95% CI: 0.84-0.86).* The NRI values were as follows: training queue: 1.16 for three-year and 1.12 for five-year OS prediction; authentication group: 1.22 for three-year and 1.21 for five-year OS prediction. The IDI values were as follows: training cohort: 0.25 for three-year and 0.21 for five-year OS prediction; validation cohort: 0.27 for three-year and 0.21 for five-year OS prediction. The DCA diagram showed that the nomogram was superior in predicting the three-year and five-year trends.

**Conclusions:**

Our nomogram can be used to forecast the survival of patients with stage IV TC.

## Introduction

Thyroid cancer (TC) is an epidemic in America, and its incidence has grown faster than that of any other cancer since the 1990s (1). An estimated 44,280 new cases occurred in men and women in 2020, with a mortality rate of 4.97%, and this has aroused substantial public concern (2). In the meantime, many patients often develop long-distance metastasis (DMs) or lymphatic metastasis, which in turn leads to a high mortality rate. TC has been classified into four phases, among which stage IV TC varies according to the clinical subtype, including anaplastic carcinoma, medullary thyroid carcinoma (MTC), papillary carcinoma (PTC) and follicular carcinoma (FTC) (3–5). In fact, studies have indicated that TC should be evaluated independently rather than analysing TC without recognizing its pathological type. In fact, all these studies indicate that TC should be studied independently rather than analysing TC without recognizing pathological type (6, 7). Likewise, 5-20% of TC patients might develop DMs resulting in high mortality, which suggests that precise assessment of their prognosis is crucial. At present, there is no individualized model to predict the prognosis of stage IV TC patients (8).

The American Joint Committee on Cancer (AJCC) staging system is the most commonly used tool for evaluating the prognosis of patients with TC. However, this assessment system has many limitations, including low accuracy, disregard of sociodemographic and clinicopathological characteristics (such as time of life, therapy or marital status), and poor performance in predicting individual survival outcomes (9, 10). In conclusion, an individual prediction model is essential for patients with stage IV TC.

Nomograms have been widely used as predictive methods in oncology in recent years. After integrating demographic and clinicopathological characteristics, the nomogram model could be more accurate and personalized than the conventional TNM staging system, and it is convenient for clinicians to predict the prognosis of patients. In our study, we used the Surveillance, Epidemiology, and End Results (SEER) database to establish a nomogram model to predict the prognosis of TC patients (11).

## Materials and Methods

### Data Resource

The recent version of the SEER 18 registries Custom Data (with additional treatment fields) was used as the data source for the present population-based investigation. This database consists of 18 population-based cancer registries and covers approximately 26% of the US population across several geographic regions (12). SEER*-STAT Software version 8.3.9 (Https://seer.cancer.gov/seerstat/) (Information Management Service, Inc. Calverton, MD, USA) was used to generate the case listing. All of the procedures were excluded from the approved guidelines. Informed patient consent was not required to access or use SEER data.

### Patients Cohort

**Figure 1:**
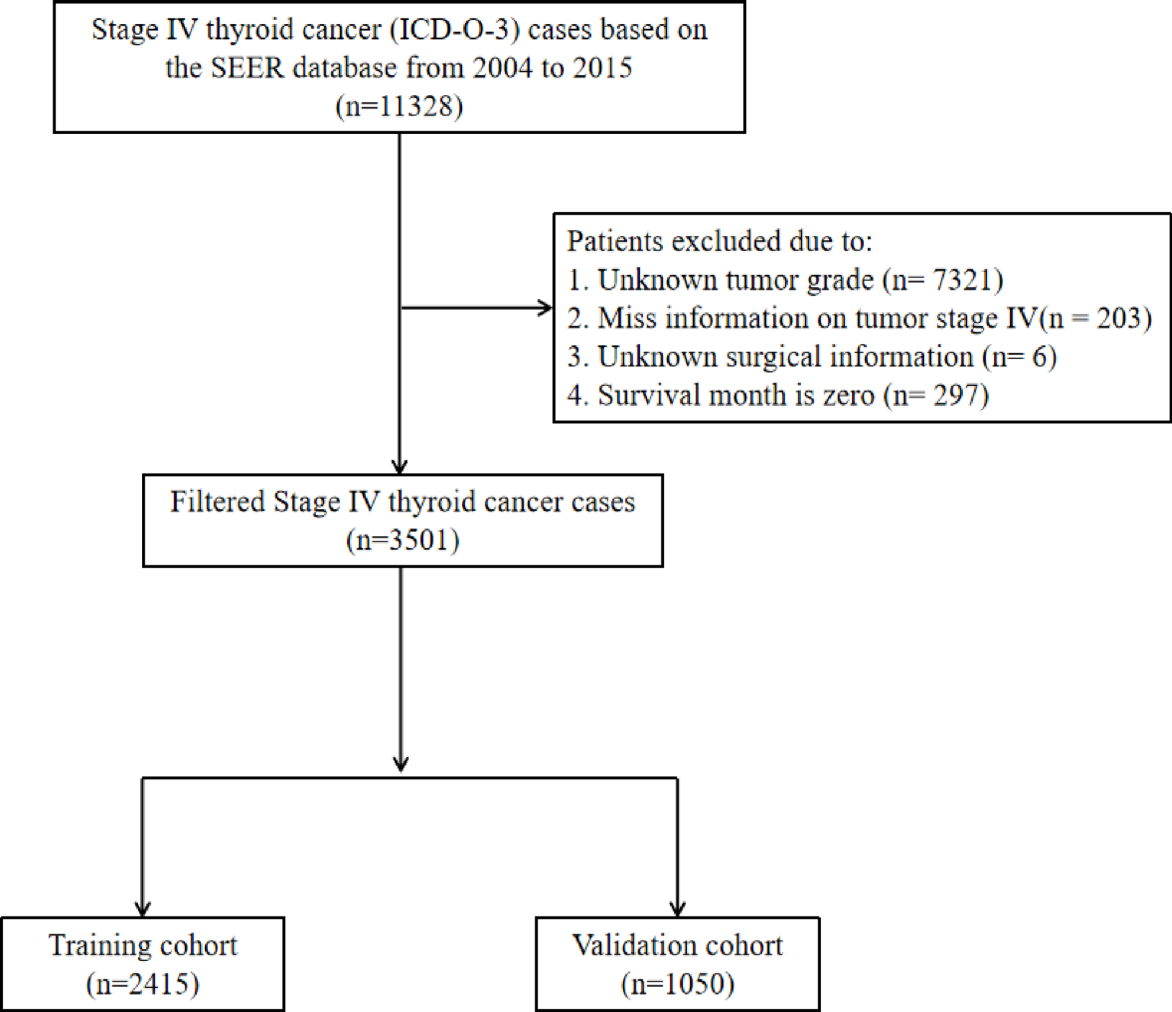
The entire screening process of Patients cohort.

Patients diagnosed with TC participated in the study, and the median survival for the patients equalled 25 months. Patients were included according to the following standards: 1) active follow-up to ensure reliable patient status; 2) TNM (American Joint Committee on Cancer, AJCC 6th) stage IV. The criteria for dismissal were as follows: 1) AJCC IV stage and tumour grade are unknown; 2) unknown operation information; and 3) the survival months is zero. After the preliminary filter, 3501 patients with TC were extracted in our cohort. In our study, the demographic and treatment features of patients were confirmed, including ethnicity year of life, time of diagnosis, civil state, operation, radiotherapy and chemotherapy. Tumour specialities consisted of tumour histology, tumour grading, AJCC staging, and prior cancer history. **Figure 1** shows the entire screening process.

### Endpoint and Statistical Analysis

Overall survival (OS) is considered the time lag between the date of diagnosis and the date of death due to any reason. We used descriptive statistics to calculate demographic and clinical variables, Student’s t test was used to describe continuous variables, and the chi-squared test was used to describe the classified variables. In survival analysis, univariate and multivariate Cox regression analyses were carried out, and the meaningful variables in univariate analysis were included in the multivariate analysis. *P*<0.05 was considered a self-governed risk factor.

Based on the above independent risk factors, a nomogram model was set up. Nomograms estimate probabilities of three and five years. The discriminant ability was evaluated by the harmony index (C index), and the calibration ability was evaluated by a calibration graph. The C-index was on a scale of 0.5 to 1.0, where 0.5 represents random opportunity and 1.0 indicates an exact accouplement. Generally, a C-index greater than 0.7 indicates a reasonable estimation. The improvement of the nomogram compared with the AJCC TNM system was evaluated by the net weight classification index (NRI), comprehensive discriminant rate improvement (IDI) and decision curve analysis (DCA). The NRI and IDI were used to assess improvements in prognosis forecasting and measure the usefulness of a new model (13, 14). DCA is a measurement for assessing the clinical benefit of alternative models and has been applied to nomograms by quantifying net benefits at different threshold probabilities (15, 16). *P values* were two-tailed, and *P* < 0.05 indicated statistical significance. In addition, the above data are analyzed by R Studio.

## Results

### Baseline Characteristics

Our cohort collected 3501 patients, and the patients were split into two groups: Group 1 was defined as the training group, and Group 2 was defined as the validation group (ratio: 7:3). The median follow-up equalled 25 months [quartile range (IQR): 6-60] in the whole population, 25 months (IQR: 6-60) in the training cohort, and 25 months (IQR: 5-59) in the validation cohort. In the whole population, training and verification teams, the mean ages of patients with TC were 64.5 (± 12.8), 64.5 (± 12.7) and 64.5 (± 12.9) years, respectively. Females(56.5%) and married patients (60.8%) comprised the majority of the cohort. Meanwhile, the whole population had a relatively low rate of moderate (11.9%) and poorly differentiated (16.9%) MTCs. As for histology, MTCs only account for a low ratio of MTCs (3.1%). Moreover, 48.2% of them had tumour stage IVA, and 81.9% of patients had no prior cancer history. In addition, 82.5% and 61.5% of the population received surgery and radiotherapy, respectively, while only 20.2% of patients received chemotherapy. There was no statistical difference between the training and validation cohorts regarding demographic and clinical characteristics (*P* > 0.05), except tumour histology. Detailed information on these TC patients is shown in **Table 1**.

**Table 1:** The baseline characteristics of patients in the SEER database.

Characteristics	All cohort		Training cohort		Validition cohort		P-value
	N	%	N	%	N	%	
	3501	100	2451	70.01	1050	29.99	
**Age(mean±SD)**	64.5 (±12.8)		64.5 (±12.7)		64.5 (±12.9)		0.98
**Race**							0.58
Black	222	6.3	162	6.6	60	5.7	
Other	487	13.9	337	13.7	150	14.3	
White	2792	79.7	1952	79.6	840	80	
**Sex**							0.14
Female	1979	56.5	1365	56.7	614	58.5	
Male	1522	43.5	1086	44.3	436	41.5	
**Grade**							0.97
Grade I	1211	34.6	780	35.5	341	32.5	
Grade II	417	11.9	288	11.8	129	12.3	
Grade III	592	16.9	412	16.8	180	17.1	
Grade IV	1281	36.6	881	35.9	400	38.1	
**Histology**							0.02
Anaplastic carcinoma	790	22.6	531	21.7	259	24.7	
Follicular carcinoma	179	5.1	140	5.7	39	3.7	
Medullary carcinoma	108	3.1	81	3.3	27	2.6	
Other	418	11.9	282	11.5	136	13	
Papillary carcinoma	2006	57.3	1417	57.8	589	56.1	
**AJCC stage**							0.95
IVA	1688	48.2	1179	48.1	509	48.5	
IVB	803	22.9	561	22.9	242	23	
IVC	1010	28.8	711	29	299	28.5	
**Surgery**							0.11
No	611	17.5	411	16.8	200	19.0	
Yes	2890	82.5	2040	83.2	850	81	
**Radiotherapy**							0.14
No	1347	28.5	923	37.3	424	40.4	
Yes	2154	61.5	1528	62.3	626	59.6	
**Chemotherapy**							0.22
No	2794	79.8	1970	80.4	824	78.5	
Yes	707	20.2	481	19.6	226	21.5	
**cancer history**							0.66
Yes	634	18.1	449	18.3	185	17.6	
No	2867	81.9	2002	81.7	865	82.4	
**Marital status**							0.53
Married	2128	60.8	1498	61.1	630	60	
Single	1263	36.1	881	35.9	382	36.4	
Unknown	110	3.1	72	2.9	38	3.6	

SEER, Surveillance, Surveillance, Epidemiology, and End Results; AJCC, American Joint Committee on Cancer.

### Nomogram Variable Screening Based on Univariate and Multivariate Cox Regression Analysis

We carried out univariate and multivariate analyses sequentially (**Table 2**), and the independent prognostic factors with a significant influence on OS were selected. Univariate analysis showed that 10 variables, such as ethnic lines, time of year, tumour grading, tumour histology, tumour staging, operation, radiotherapy, chemotherapy, prior cancer history and marital status, showed positive statistical significance.

**Table 2:** Cox proportional hazard model of OS.

Characteristics	Univariate analysis			Multivariate analysis		
	Hazard ratio	95% CI	*P*-value	Hazard ratio	95% CI	*P*-value
**Race**						
Black		As reference			As reference	
Other	0.68	0.52-0.88	0.003	0.90	0.69-1.18	0.46
White	0.76	0.61-0.94	0.01	0.91	0.73-1.13	0.37
**Age**	1.05	1.04-1.06	<0.001	1.03	1.02-1.04	<0.001
**Sex**						
Female	As reference					
Male	0.94	0.84-1.05	0.290			
**Grade**						
I		As reference			As reference	
II	1.43	1.08-1.91	0.01	1.27	0.95-1.69	0.11
III	5.51	4.5-6.7	<0.001	3.24	2.61-4.02	<0.001
IV	11.05	9.24-13.21	<0.001	5.20	4.17-6.48	<0.001
**Histology**						
Anaplastic carcinoma	As reference				As reference	
Follicular carcinoma	0.24	0.19-0.31	<0.001	0.56	0.42-0.75	<0.001
Medullary carcinoma	0.22	0.16-0.31	<0.001	0.54	0.37-0.77	<0.001
Other	0.51	0.44-0.6	<0.001	0.77	0.64-0.94	0.01
Papillary carcinoma	0.12	0.11-0.14	<0.001	0.6	0.5-0.72	<0.001
**AJCC stage**						
IVA		As reference			As reference	
IVB	5.04	4.31-5.89	<0.001	1.99	1.68-2.35	<0.001
IVC	6.92	5.96-8.04	<0.001	3.89	3.31-4.58	<0.001
**Surgery**						
No		As reference			As reference	
Yes	0.15	0.14-0.17	<0.001	0.64	0.54-0.76	<0.001
**Radiotherapy**						
No		As reference		As reference		
Yes	0.39	0.35-0.44	<0.001	0.65	0.56-0.74	<0.001
**Chemotherapy**						
No		As reference		As reference		
Yes	3.21	2.84-3.63	<0.001	1.05	0.91-1.21	0.5
**Prior cancer history**						
Yes		As reference		As reference		
No	0.75	0.65-0.86	<0.001	0.91	0.79-1.05	0.19
**Marital status**						
Married		As reference		As reference		
Single	1.48	1.32-1.66	<0.001	1.21	1.07-1.36	0.002
Unknown	0.96	0.67-1.38	0.82	0.83	0.58-1.21	0.34

SEER, Surveillance, Surveillance, Epidemiology, and End Results; AJCC, American Joint Committee on Cancer; ER, estrogen receptor; PR, progesterone receptor; OS, Overall Survival; BCS, breast conserving surgery; SM, simple mastectomy; RM, radical mastectomy.

Multivariate Cox proportional risk regression analysis showed that time of life, tumour grading, tumour histology, tumour staging, surgery, radiotherapy and marital status were significantly correlated with OS in the study. In conclusion, the prognostic variables with statistical significance in univariate and multivariate analyses were independent predictive factors for stage IV TC patients.

### Nomogram Construction and Validation

**Figure 2:**
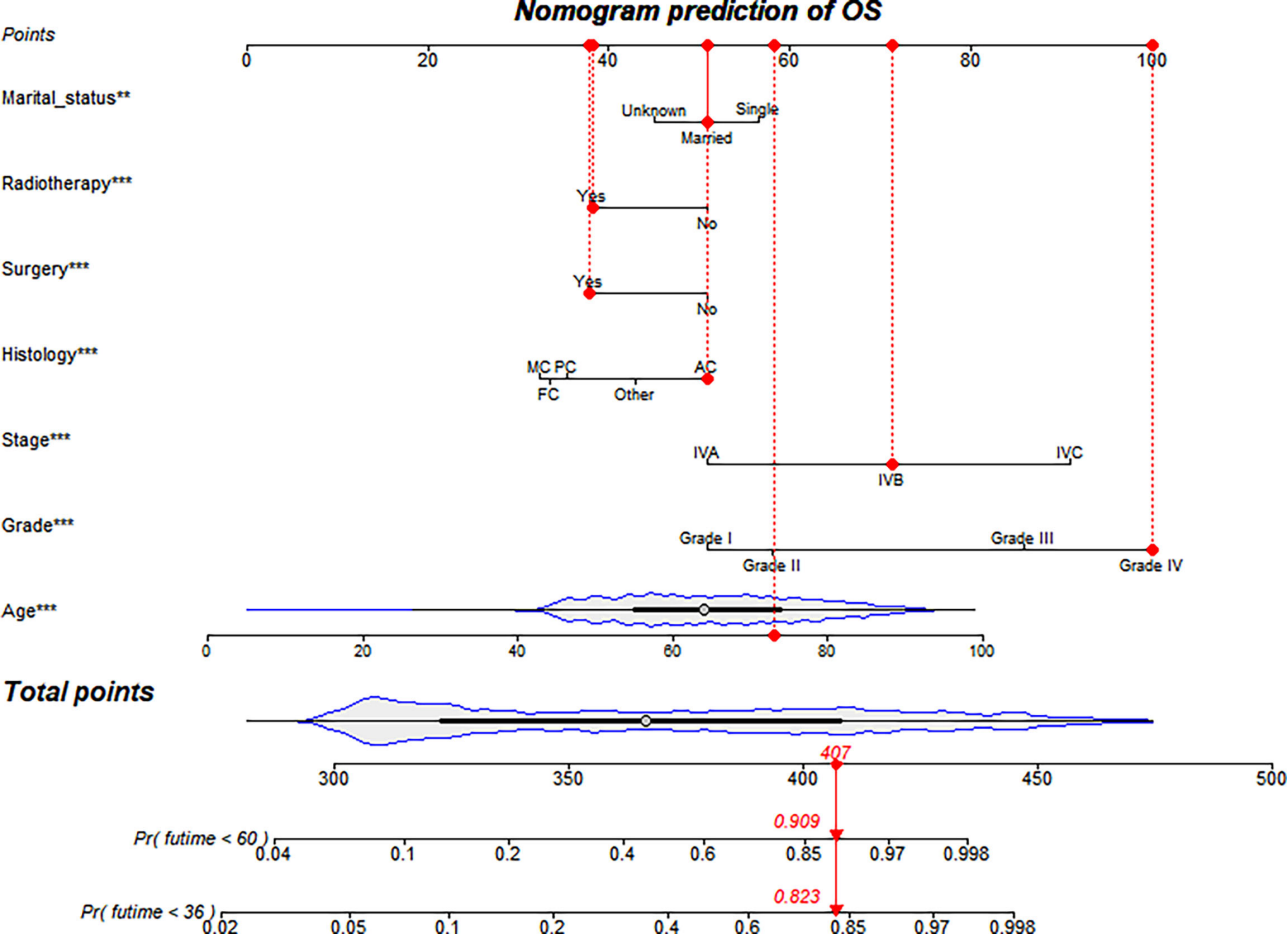
nomogram prediction of OS.

Based on screening variables, the nomogram model constructed by Cox proportional hazard regression was used to predict the OS of stage IV TC patients at 3 years and 5 years. As shown in **Figure 2**, screening variables pointed to a score, and a total score was obtained by adding up all of the scores. A nomogram can be used to predict the survival probability of a given patient. Furthermore, most patients in our study had total cumulative points ranging from 300 to 450 (**Figure 2**).

**Figure 3:**
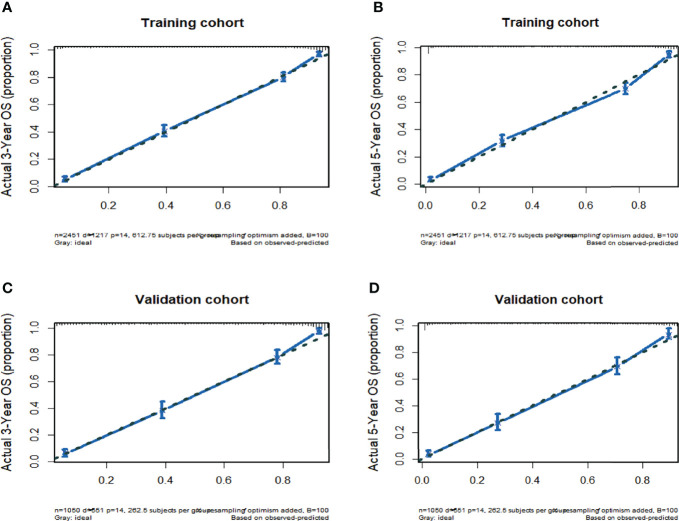
**(A)** Nomogram-Predicted probability of 3-Year OS. **(B)** Nomogram-Predicted probability of 5-Year OS. **(C)** Nomogram-Predicted probability of 3-Year OS. **(D)** Nomogram-Predicted probability of 5-Year OS.

The C-index value equalled 0.86 (95% CI: 0.85-0.87) in the training queue and 0.85 (95% CI: 0.84-0.86) in the validation queue (**Table 3**). The calibration curve of the nomogram also showed high consistency between the nomogram prediction and operating system observed in the training and verification queue for three years and five years (**Figures 3A–D**). In conclusion, the nomogram for TC patients had great discriminative and calibrating abilities.

**Table 3:** C-index, NRI and IDI of the nomogram and AJCC-TNM stage system in OS prediction for stage IV TC patients.

	Training cohort			Validation cohort		
	Estimate	95% CI	P-value	Estimate	95% CI	P-value
NRI (vs. AJCC-TNM stage system)						
For 3-year OS	1.16	1.09-1.23		1.22	1.06-1.34	
For 5-year OS	1.12	1.05-1.19		1.21	1.04-1.31	
IDI (vs. AJCC-TNM stage system)						
For 3-year OS	0.25	0.21-0.27	<0.001	0.27	0.22-0.31	<0.001
For 5-year OS	0.21	0.18-0.24	<0.001	0.24	0.19-0.28	<0.001
C-index						
The nomogram	0.86	0.85-0.87		0.85	0.84-0.86	
AJCC-TNM stage system	0.72	0.71-0.73		0.71	0.7-0.72	

AJCC, American Joint Committee on Cancer; OS, Overall survival; TC, Thyroid cancer

### Nomogram Clinical Value to Compare With AJCC-TNM System Stage

The NRI and IDI were used to compare the accuracy between the nomogram and the AJCC-TNM stage (**Table 3**). In the training cohort, the NRI values for the three-year and five-year OS were 1.16 (95% CI: 1.09-1.23) and 1.12 (95% CI: 1.05-1.19), respectively; the IDI values for the three-year and five-year OS equalled 0.25 (95% CI: 0.21-0.27, P < 0.001) and 0.21 (95% CI: 0.18-0.24, P<0.001), respectively. The validation cohort indicated that the NRI values for the three-year and five-year OS were 1.22 (95% CI: 1.06-1.34) and 1.21 (95% CI: 1.04-1.31), respectively, and the IDI values for the three-year and five-year OS rates were 0.27 (95% CI: 0.22-0.31, P<0.001) and 0.24 (95% CI: 0.19-0.28, P<0.001), respectively.

**Figure 4:**
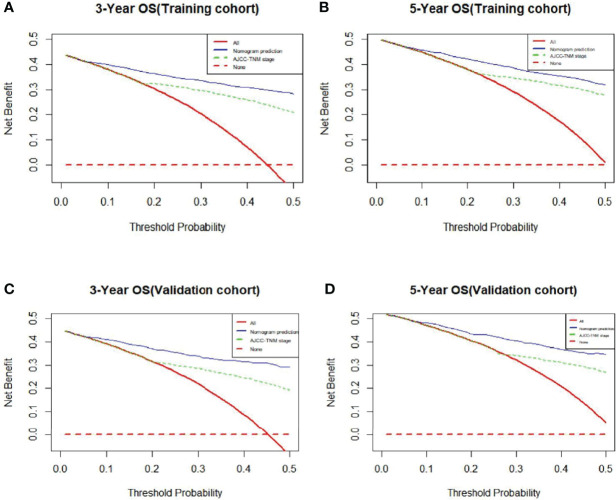
**(A)** 3-Year OS. **(B)** 5-Year OS. **(C)** 3-Year OS. **(D)** 5-Year OS.

The clinical benefits of the nomogram were compared with those of the AJCC-TNM system stage. The DCA chart shows that the nomogram had a certain advantage in predicting 3-year and 5-year OS, as it increased net benefits more than the AJCC-TNM system stage for almost all threshold probabilities in both the training and validation cohorts and with both the treat-all-patients scheme and the treat-none scheme (**Figures 4A–D**). In summary, the abovementioned results indicated that the nomogram model could increase precision and reliability for OS prediction compared with the AJCC-TNM staging system.

## Discussion

Stage IV TC is a tumour with a relatively poor prognosis, but it lacks a precise and efficient instrument to forecast prognosis in individual patients in clinical practice, except for the AJCC TNM staging system. On the basis of the records of 3501 patients from the SEER database, our team put up a nomogram model to forecast the three-year and five-year OS of patients with stage IV TC. Seven variables were selected by clinical significance to construct and validate the capability of the model, which could provide the basis for future clinical decisions. Measured by range along nomogram scales, age was the most important prognostic factor, followed by tumour grading, tumour staging, tumour histology, surgery, radiotherapy and marital status. This is the first study to predict the prognosis of patients with stage IV TC, and the population-based nomogram model showed the greatest results.

In our study, prognosis was estimated by OS, which is a common and objective index for patients with stage IV TC. Univariate Cox regression analysis showed that ethnic lines, time of life, tumour grading, tumour histology, tumour staging, operation, radiotherapy, chemotherapy, past cancer history and marital status were significantly related to OS. To decrease the estimation bias and further confirm the independent prognostic factors on OS for patients with TC, multivariate Cox regression analysis was performed.

After readjusting the demographic, clinicopathological and therapeutic variables, we discovered that time of life, tumour grading, tumour histology, tumour staging, operation, radiotherapy and marital status were still significantly related to OS. Through univariate and multivariate Cox regression analysis, the above 7 variables were selected to build the prediction model.

In conclusion, the nomogram model can be used to predict the survival outcome of various cancers, which integrates clinical and demographic factors to evaluate the risk of specific diseases (10, 17–20). Traditionally, the AJCC-TNM staging system has been the primary choice to predict prognosis and make clinical decisions. However, the prognosis of patients at the same stage is often different because the AJCC-TNM staging system does not comprehensively consider various variables, such as clinical characteristics, treatment methods and sociodemographic characteristics. Therefore, we compared the nomogram, which involves more variables, with the conventional AJCC-TNM staging system. The NRI value and IDI value of the nomogram versus the TNM staging system suggested that the nomogram prediction model had better predictive capability than the TNM staging system alone. Furthermore, DCA curves demonstrated that our model forecasted survival outcome with better clinical value and utility than the conventional staging system. In the validation cohort, the results could also be replicated favourably. In conclusion, our nomogram could provide accurate and individual predictions of OS in patients with TC.

Since many patients with stage IV TC have concerns about balancing the risk of surgery and the chance for a longer life, there is great need to build a scoring system for reference. Using this model, we could easily and precisely predict individual patients’ overall death probability at certain time points.

First, different variables pointed to a score according to the top scale, and we drew a vertical line from each prognostic factor to obtain the corresponding points. Second, we obtained a total score by summing all of the scores. Finally, we drew a vertical line from the total points scale to the 3- and 5-year overall death scale to obtain the estimated probabilities of death, and from this we obtained the OS rate. For instance, 73-year-old married patients underwent surgery and radiotherapy, with anaplastic carcinoma and grade II and stage IVB tumours. Since we can estimate from the nomogram graph that the 3-year and 5-year overall death probabilities are 82.3% and 90.9%, the 3-year and 5-year OS rates are 17.7% and 9.1%, respectively.

Although the nomogram performed well, our study did have some limitations, as shown below. First, the nomogram was based on a retrospective study, which could not prove causation and result due to selection bias. Second, we were unable to exclude the impact of potential confounders, such as family history, complications, health conditions, and patient anxiety, which were not covered in the SEER database. Last, a *P* value < 0.05 was used to determine statistical significance, and no adjustment was made for multiple analyses. The probability of false rejection of invalid hypotheses may have exceeded 0.05. Multicentre clinical trials are needed to evaluate the external utility of our nomogram.

## Conclusion

Our study aimed to construct a nomogram for predicting prognosis in patients with stage IV TC based on data from the SEER programme. Given its favourable clinical utility and accurate prognosis prediction in comparison with the conventional TNM staging system, our nomogram can be used to predict the survival of patients with stage IV TC. However, multicentre clinical validation is also required to evaluate the external utility of our histograms.

## Data Availability Statement

The original contributions presented in the study are included in the article/supplementary material. Further inquiries can be directed to the corresponding authors.

## Author Contributions

TY and TH analysed the data. TH drafted the manuscript. TY generated the figure. MZ and QH edited the manuscript. All authors contributed to the article and approved the submitted version.

## Funding

This study was supported by grants from the Hunan innovative province construction project (Grant No. 2019SK2211), the National Natural Science Foundation of Hunan province (Grant No. 2020JJ4833), Key research and development project of Hunan Province (Grant No. 2020SK2089).

## Conflict of Interest

The authors declare that the research was conducted in the absence of any commercial or financial relationships that could be construed as a potential conflict of interest.

## Publisher’s Note

All claims expressed in this article are solely those of the authors and do not necessarily represent those of their affiliated organizations, or those of the publisher, the editors and the reviewers. Any product that may be evaluated in this article, or claim that may be made by its manufacturer, is not guaranteed or endorsed by the publisher.

## References

[1] Davies L Welch HG. Current Thyroid Cancer Trends in the United States. JAMA Otolaryngol Head Neck Surg (2014) 140(4):317–22. doi: 10.1001/jamaoto.2014.1 24557566

[2] Gardsvoll H Kriegbaum MC Hertz EP Alpizar-Alpizar W Ploug M. The Urokinase Receptor Homolog Haldisin is a Novel Differentiation Marker of Stratum Granulosum in Squamous Epithelia. J Histochem Cytochem (2013) 61(11):802–13. doi: 10.1369/0022155413501879 PMC3808577 23896969

[3] Hirsch D Levy S Tsvetov G Gorshtein A Slutzky-Shraga I Akirov A. Long-Term Outcomes and Prognostic Factors in Patients With Differentiated Thyroid Cancer and Distant Metastases. Endocr Pract (2017) 23(10):1193–200. doi: 10.4158/EP171924.OR 28704099

[4] Li M Trivedi N Dai C Mao R Wang Y Ning Y. Does T Stage Affect Prognosis in Patients With Stage Iv B Differentiated Thyroid Cancer? Endocr Pract (2019) 25(9):877–86. doi: 10.4158/EP-2019-0051 31170365

[5] Shaha AR Migliacci JC Nixon IJ Wang LY Wong RJ Morris LGT. Stage Migration With the New American Joint Committee on Cancer (AJCC) Staging System (8th Edition) for Differentiated Thyroid Cancer. Surgery (2019) 165(1):6–11. doi: 10.1016/j.surg.2018.04.078 30415873 PMC6309303

[6] Chen Z Ruan J Yao Y Wen L Mao Z Chen S. A Comparison of the Seventh and Eighth Editions of the AJCC Staging Systems to Predict Recurrence in Papillary Thyroid Microcarcinoma. Ann Surg Oncol (2021) 28(11):6564-6571. doi: 10.1245/s10434-021-09596-6 33521903

[7] Lechner MG Bernardo AC Lampe A Praw SS Tam SH Angell TE. Changes in Stage Distribution and Disease-Specific Survival in Differentiated Thyroid Cancer With Transition to American Joint Committee on Cancer 8th Edition: A Systematic Review and Meta-Analysis. Oncologist (2021) 26(2):e251–60. doi: 10.1634/theoncologist.2020-0306 PMC7873343 32864832

[8] Schmid KW. [Lymph Node and Distant Metastases of Thyroid Gland Cancer. Metastases in the Thyroid Glands]. Pathologe (2015) 36 Suppl 2:171–5. doi: 10.1007/s00292- 015-0071-6 10.1007/s00292-015-0071-6 26357953

[9] Fang C Wang W Feng X Sun J Zhang Y Zeng Y. Nomogram Individually Predicts the Overall Survival of Patients With Gastroenteropancreatic Neuroendocrine Neoplasms. Br J Cancer (2017) 117(10):1544–50. doi: 10.1038/bjc.2017.315 PMC5680463 28949958

[10] Mahar AL Compton C Halabi S Hess KR Weiser MR Groome PA. Personalizing Prognosis in Colorectal Cancer: A Systematic Review of the Quality and Nature of Clinical Prognostic Tools for Survival Outcomes. J Surg Oncol (2017) 116(8):969–82. doi: 10.1002/jso.24774 PMC5760443 28767139

[11] Ni X Ma X Qiu J Zhou S Cheng W Luo C. Development and Validation of a Novel Nomogram to Predict Cancer-Specific Survival in Patients With Uterine Cervical Adenocarcinoma. Ann Transl Med (2021) 9(4):293. doi: 10.21037/atm-20-6201 33708920 PMC7944266

[12] Wingo PA Jamison PM Hiatt RA Weir HK Gargiullo PM Hutton M. Building the Infrastructure for Nationwide Cancer Surveillance and Control–a Comparison Between the National Program of Cancer Registries (NPCR) and the Surveillance, Epidemiology, and End Results (SEER) Program (United States). Cancer Causes Control (2003) 14(2):175–93. doi: 10.1023/A:1023002322935 12749723

[13] Cook NR Paynter NP. Comments on 'Extensions of Net Reclassification Improvement Calculations to Measure Usefulness of New Biomarkers' by M. J. Pencina, R. B. D'Agostino S.R. And E. W. Steyerberg. Stat Med (2012) 31(1):93–95; author reply 96-97. doi: 10.1002/sim.4209. Ep ub 2011 Feb 23 21344474

[14] Uno H Tian L Cai T Kohane IS Wei LJ. A Unified Inference Procedure for a Class of Measures to Assess Improvement in Risk Prediction Systems With Survival Data. Stat Med (2013) 32(14):2430–42. doi: 10.1002/sim.5647 PMC3734387 23037800

[15] Fitzgerald M Saville BR Lewis RJ. Decision Curve Analysis. JAMA (2015) 313(4):409–10. doi: 10.1001/jama.2015.37 25626037

[16] Vickers AJ Elkin EB. Decision Curve Analysis: A Novel Method for Evaluating Prediction Models. Med Decis Making (2006) 26(6):565–74. doi: 10.1177/0272989X06295361 PMC2577036 17099194

[17] Kong J Zheng J. A nomogram for individualized estimation of survival among adult patients with adrenocortical carcinoma after surgery: a retrospective analysis and multicenter validation study. Cancer Commun (Lond) (2019) 39(1):80. doi: 10.1186/s40880-019-0426-0. PMC6882048 31775884

[18] Wang C Yang C Wang W Xia B Li K Sun F. A Prognostic Nomogram for Cervical Cancer after Surgery from SEER Database? J Cancer (2018) 9(21):3923-3928. doi: 10.7150/jca.26220 30410596 PMC6218784

[19] Tang J Jiang S Gao L Xi X Zhao R Lai X. Construction and Validation of a Nomogram Based on the Log Odds of Positive Lymph Nodes to Predict the Prognosis of Medullary Thyroid Carcinoma After Surgery? Ann Surg Oncol (2021) 28(8):4360-4370. doi: 10.1245/s10434-020-09567-3 33469797

[20] Tang J Tian Y Xi X Ma J Li H Wang L. A novel prognostic model based on log odds of positive lymph nodes to predict outcomes of patients with anaplastic thyroid carcinoma after surgery? Clin Endocrinol (Oxf) (2022). doi: 10.1111/cen.14729 35355304

